# Self-reported compliance with routine prenatal medications by pregnant women in a tertiary hospital in Enugu State, Nigeria

**DOI:** 10.18332/ejm/130595

**Published:** 2020-12-21

**Authors:** Adaobi L. Obiekwu, Chisom J. Mbadugha, Chinenye J. Anetekhai, Nonyelum G. Isife, Christianah O. Kotoye

**Affiliations:** 1 Department of Nursing Sciences, Faculty of Health Sciences and Technology, University of Nigeria, Nsukka, Nigeria; 2 Enugu State University of Science and Technology Teaching Hospital Parklane, Enugu State, Nigeria

**Keywords:** compliance, pregnant women, routine medications

## Abstract

**INTRODUCTION:**

Compliance to routine antenatal medications increases a woman’s chances of achieving a normal pregnancy and having a healthy baby. However, non-compliance is a commonly encountered problem in developing countries including Nigeria. This study investigated compliance to routine antenatal medications as reported by pregnant women receiving antenatal care in a tertiary hospital in Enugu State, Nigeria.

**METHODS:**

A cross-sectional descriptive survey research design was adopted for the study while simple random sampling technique was used to select the 354 pregnant women at Enugu state teaching hospital. A validated structured questionnaire (α=0.72) was utilized for data collection. Analysis of data involved descriptive and inferential statistics.

**RESULTS:**

Only 32% of pregnant women had a good compliance to their routine antenatal drugs while forgetfulness (27.2%) and vomiting as a side effect of the drug (25.7%) were the major barriers to compliance with routine drugs. Furthermore, there was a statistically significant association between respondents’ compliance with antenatal drugs and number of children as well as level of income (p<0.001). No statistically significant association was found between education level and compliance.

**CONCLUSIONS:**

Nurses and other health workers should support pregnant women to set reminders for taking their medications and prescribe alternative dosage forms or products that will not induce vomiting. In addition, antenatal education should emphasize the benefits of adherence to routine taking of medications at every stage of pregnancy and the possible negative consequences of non-compliance.

## INTRODUCTION

Pregnancy and birth of a baby are important stages in life^1^, welcomed by families, relatives and the community with much happiness. However, the period of pregnancy is a period of great demand for nourishment for the mother on whom the foetus is dependent upon for its nutrients. Thus, proper dietary intake of energy foods and micronutrients is essential for adequate nourishment of mother and foetus^2^.

Although it is argued that diet alone, if sufficiently fortified, can meet nutritional demands during pregnancy, some authors noted that many women, especially in low- and middle-income countries, have inadequate diets that are lacking in nutrients and micronutrients necessary for maintaining good health^3^. These deficiencies become more evident during gestation as the baby makes its own demands via the placenta. The exacerbation of the nutrient deficiency can influence the overall health of both mother and foetus and may result in adverse effects^4^.

Globally, iron-deficiency anaemia is the commonest micronutrient deficiency^5^. According to Stevens et al.^6^, the prevalence of anaemia in pregnancy was 38% worldwide, which is equivalent to 32 million pregnant women in the world. It was also noted that West Africa, Central Africa and Asia account for the majority of these women. The adverse events associated with deficiency of iron and other nutrients required for normal metabolism and health of the mother and foetus contribute to maternal mortality^7^. An association has been found between iron deficiency in pregnancy and low birthweight^8^. It is also thought that there is an association between vitamin deficiency and the risk of spontaneous abortion^9^.

Considering the importance of adequate nutrients to both the mother and the child, it is necessary for pregnant women to supplement their diet with vitamins in order to meet the daily recommended amounts of micronutrients. As part of routine prenatal care, the World Health Organization recommends iron and folic acid supplements^1^. Also, Balogun et al.^9^ suggest that micronutrient supplements should also be taken by women planning a baby in the near future. These have been linked with a reduced risk of maternal anaemia, congenital abnormalities (such as neural tube defects, orofacial deformities, foetal heart anomalies) and low birthweight infants^8,10,11^.

In line with the recommendations, at each antenatal visit in most developing countries, prenatal medication is prescribed and given to the pregnant women throughout their gestational period. These medications and supplements include iron, folic acid, vitamin B complex, vitamin C, malaria drug for prophylaxis, tetanus toxoid immunization, etc. However, it has been reported in the literature that compliance is low^2^. Non-compliance to antenatal medication as well as its adverse effects is a commonly encountered problem in developing countries, including Nigeria. For instance, it was reported that the compliance of pregnant women to antenatal medication in developing countries was low (28.7%)^12^. It has also been found that about 58 million women in developing countries are anaemic during pregnancy and this situation was likely the result of non-compliance of the pregnant women with antenatal medication^11,13^. Additionally, studies^13,14^ have suggested that babies born to mothers who do not comply with the taking of antenatal medications are more likely to be born prematurely (before the 37th week of pregnancy), have a low birthweight, have problems with iron levels and a poorer performance in mental ability tests; they are also at increased risk of infection, heart and lungs problems, restless leg syndrome, etc.

Good compliance with antenatal medication increases a pregnant woman’s chance of giving birth normally and helps to achieve a global reduction in maternal and child morbidity and mortality. Compliance, in this regard, refers to the extent to which the behaviour of the patient coincides with medical or health advice. It may be classified either as complete compliance, partial compliance, non-compliance or there may be over compliance^5^. Many factors may play a role whether patients comply with their therapy and pregnancy may present unique challenges to pregnant women. Some typical barriers influencing medication compliance in pregnancy mentioned in the literature^5,15^ include expected side effects from the medication, cost of the drug, taste of the drug, forgetfulness, disappearance of the complaints for which the medication was prescribed, unavailability of the medication, fear of having a big baby, etc.

Therefore, there is a need to investigate adherence and possible reasons for non-adherence to the taking of antenatal medication. Results of a study that investigated the prevalence of anaemia in pregnancy in a similar setting found a 64.1% prevalence^16^. A study on the epidemiology of central nervous system congenital anomalies in children found that spinae bifida cystica was the commonest problem and could be linked to mothers who did not take prenatal folic acid supplements^17^. Furthermore, anecdotal reports of women rejecting their routine antenatal medication motivated the researchers to study pregnant women’s compliance and to identify possible reasons for non-compliance. In the light of the above, the central aim of this study was to assess compliance with taking routine antenatal medications and the possible barriers to compliance.

## METHODS

### Study design and setting

The study employed a descriptive cross-sectional design to survey pregnant women attending an antenatal clinic in Enugu State University Teaching Hospital, Parklane, Enugu North Local Government Area, Enugu State, Nigeria. It is a government owned tertiary institution with a large population of women utilizing antenatal services mainly because of its proximity and accessibility for residents of the Enugu metropolis and the availability of specialist doctors and nurses. Also, it has been very popular because of the free maternal and child health services.

### Sample and sampling technique

The population for the study comprised pregnant women attending the antenatal clinic in Enugu State University Teaching Hospital, Parklane, Enugu state. From the records available at the antenatal clinic, an average of 1145 pregnant women visit the clinic monthly, with an attendance of about 58 women for each working day. A sample size of 359 was determined using power analysis. In order to select 359 pregnant women over the 12 days that data were collected, three times a week over a 4-week period, a simple random sampling method was used to select 30 women each day from the number that attended. Since most women visit once each month, the researchers ensured that those who had filled in the questionnaire on previous occasions were excluded. Only pregnant women aged ≥20 years, who have made at least two antenatal visits and were willing to participate were included in the study. Ethics approval to conduct the study was given by the ethics review board of Enugu State University Teaching Hospital and administrative permit was obtained from the Head of the Nursing Services Unit and the Chief Nursing Officer of the antenatal clinic before accessing the participants. Oral consent was also obtained from each one of them before the questionnaires were administered. Confidentiality and anonymity of the information given was also maintained.

### Instrument for data collection

The instrument that was used for data collection was a structured questionnaire developed based on the objectives of the study. The questionnaire consisted of closed-ended questions that were arranged in three sections. Section A had seven questions that elicited responses on the demographic data of the pregnant women. Section B comprised questions that elicited responses on the level of compliance of the pregnant women to their prescribed antenatal medication. It had five items for which a respondent indicated the frequency of occurrence, while Section C comprised questions that elicited responses on the barriers that influence compliance of the pregnant women to their prescribed antenatal medication. Each respondent with a history of missed medication selected a contributory factor to missing the drug from 12 items listed. The questionnaire was validated by two mother-and-child health specialists to rule out ambiguities and ensure that the questions addressed the objectives of the study. A pilot test was done to determine the reliability of the instrument by administering thirty-five copies of the questionnaire to pregnant women attending the antenatal clinic at the University of Nigeria Teaching Hospital, Ituku-Ozalla. The test-retest method was used and yielded a Cronbach alpha of 0.72 on computation.

### Data collection

Following approval, the respondents were approached during their visits at the antenatal clinic. The researchers utilized the waiting time at the clinic to explain the aim of the study and make clarifications based on the questions that were asked. With the aid of two trained research assistants, the questionnaires were administered to those who met the inclusion criteria and were willing to participate in the study. While some respondents were able to fill in their responses alone, the researchers assisted others to fill in the responses and cleared any confusions that arose. Completed questionnaires were collected on the spot. This was done on Mondays, Wednesdays and Fridays for a period of four weeks.

### Statistical analysis

Data analysis was completed by means of IBM statistic software (SPSS) for windows version 24. The collected data were reviewed and checked for completeness and consistency of response, sorted, categorized, and coded. Questionnaires that were incorrectly filled in were excluded from further analysis. The data were then used for descriptive statistics to generate frequencies, means and standard deviations (where applicable). Concerning the subjects’ compliance to antenatal medication, a score of one (1) point each was given to participants who always bought antenatal medication as prescribed, always took it as prescribed, had never missed taking their medication, never reduced the number of medications taken based on the healthcare provider’s prescription, did not take more than the prescribed medication, and participants who never missed taking their medication, otherwise the participants were given a score of zero; giving finally a score range of 0–6 points. Subjects who scored ≥4 points were grouped as having good compliance, while those scoring lower were grouped as having poor compliance to their routine medication. Simple proportion (percentage) was used to report the subjects’ responses regarding the factors that influence compliance to their routine medication. Finally, inferential statistics (hypotheses) was tested using chisquared (Non-parametric Kruskal Wallis) with two-sided p≤0.05 considered statistically significant.

## RESULTS

A total of 354 questionnaires representing 97.52% were included for analysis. Although 359 questionnaires were recovered, 5 questionnaires were incorrectly filled in and thus were excluded from the analysis. The results showed that 42.7% of the respondents were aged 26–31 years, 39.8% were aged 32–37 years (mean: 31.2 ± 4.8 years). About four-fifths (81.6%) of the subjects had completed university education, and were married in a monogamous setting (94.6%). Furthermore, employment in the civil service (38.7%) was the most common occupation among the respondents, followed by self-employment (25.7%). Also, over half (57.1%) of the respondents were living on a monthly income of ≤50000 NGN (10000 Nigerian Naira about 26 US$), and had either no living child or just one (56.7%) (Table 1).

**Table 1:** Respondents’ sociodemographic characteristics (N=354)

*Characteristics*	*Categories*	*n*	*%*
**Age** (years)	20–25	34	9.6
	26–31	151	42.7
	32–37	141	39.8
	≥38	28	7.9
**Highest education attained**	No formal education	2	0.6
	Secondary	63	17.8
	Tertiary	289	81.6
**Marital status**	Married in monogamous setting	335	94.6
	Married in polygamous setting	15	4.2
	Single	4	1.1
**Occupation**	Civil servant	137	38.7
	Self employed	91	25.7
	Employed by private sector	20	5.6
	Unemployed	57	16.1
	Student	47	13.3
	Youth corper	2	0.6
**Average monthly income** (NGN)	≤50000	202	57.1
	51000–100000	136	38.4
	101000–150000	7	2.0
	≥151000	9	2.5
**Number of children**	0	101	28.5
	1	100	28.2
	2	84	23.7
	3	51	14.4
	4	11	3.1
	≥5	7	2.0

NGN: 10000 Nigerian Naira about 26 US$.

Concerning the subjects’ compliance to antenatal medication, Table 2 shows that only 37.9% always bought antenatal medication as prescribed, 31.4% always took it as prescribed, while only 19.8% had never missed taking their medication.

**Table 2:** Antenatal drug compliance by pregnant women in a tertiary hospital in Enugu state, Nigeria (N=354)

*Variable*	*Parameters*	*n*	*%*
**Buys antenatal drugs as prescribed**	Always	134	37.9
	Sometimes	220	62.1
**Takes routine drugs as prescribed**	Always	111	31.4
	Sometimes	243	68.6
**Miss taking the drugs on some days**	Always	8	2.3
	Sometimes	276	77.9
	Never	70	19.8
**Reduces the number of drugs taken without healthcare provider’s prescription**	Always	4	1.1
	Sometimes	40	11.3
	Never	310	87.6
**Takes more than the prescribed drugs**	Sometimes	4	1.1
	Never	350	98.9
**Ever missed taking the drugs**	No	78	22.0
	Yes	276	78.0

However, 87.6% of the subjects had never reduced the number of medications taken without the healthcare provider’s prescription. Similarly, almost all of the subjects (98.9%) did not take more than the prescribed medication but 78% reported ever missing to take their medication (Table 2). Overall, only 32% of the respondents had good compliance (scored ≥4 points on the compliance questions).

Chi-squared test of independence (non-parametric Kruskal Wallis) was run to assess for an association between compliance with antenatal medication and the respondents’ education level (Table 3). Although the result revealed that compliance increased with higher education level, with subjects who had completed tertiary education demonstrating the highest level of compliance (mean rank = 181.2), and subjects without formal education showing the least compliance (mean rank = 120.0), the observed difference was not statistically significant (p=0.157). For compliance with antenatal medication and the respondents’ number of children (Table 3), the result showed that subjects who had no children showed the highest level of compliance, followed by those who had only one child, and then subjects with three children. Furthermore, compliance with antenatal medication and the respondents’ level of income, was also statistically significant.

**Table 3:** Association between compliance with antenatal medications among the respondents and some selected sociodemographic characteristics by pregnant women in a tertiary hospital in Enugu state, Nigeria

*Characteristics*	*Categories*	*N*	*Mean rank*	*Chi-squared*	*p*
**Highest education attained**	No formal education	2	120.00	χ^2^(2)=3.7	0.157
	Secondary	63	162.14		
	Tertiary	289	181.25		
**Number of children**	0	101	219.89	χ^2^(5)=57.57	<0.001
	1	100	181.95		
	2	84	134.75		
	3	51	175.53		
	4	11	120.00		
	≥5	7	120.00		
**Average monthly income** (NGN)	≤50000	202	196.23	χ^2^(3)=24.96	<0.001
	51000–100000	136	151.24		
	101000–150000	7	145.29		
	151000–200000	9	179.00		

NGN: 10000 Nigerian Naira about 26 US$. Chi-squared output (non-parametric Kruskal Wallis).

**Figure 1:**
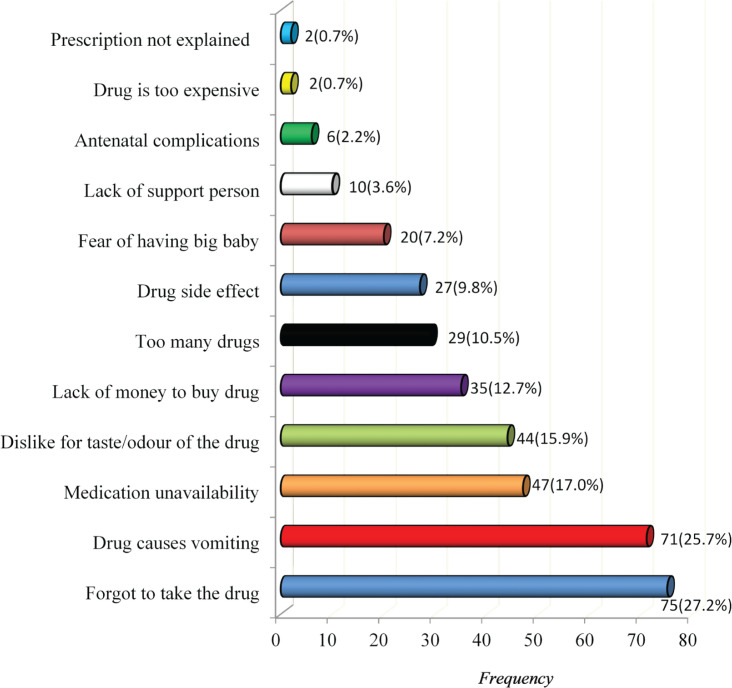
Bar chart showing the barriers to compliance with the prescribed antenatal drugs, N=276

On barriers to compliance with antenatal medication, the strongest reason why the subjects stop or miss their medication was forgetting to take the drug (27.2%) followed by reports of the medication causing them to vomit (25.7%), medication unavailability (17%), dislike of the colour, shape, taste or odour of the medication (15.9%), lack of money to purchase the medication (12.7%), among others (Figure 1).

## DISCUSSION

This study demonstrated a poor level of compliance among pregnant women, as less than half always bought their routine medications and used them as prescribed. Compliance refers to the extent to which the pregnant woman follows medical prescription and this has been a major issue in micronutrient supplementation in pregnancy^13,18^. Although a little less than two-thirds of the respondents were either primiparous or with one child and the majority also had tertiary education, it is rather surprising that compliance was still poor, as they were expected to keep to their prescription being new to the pregnancy experience. This finding is similar to that found in Kenya^19^, Malawi^20^, and South Ethiopia^21^, where compliance was still below 50%. In contrast, good compliance of over 90% was found in South Africa^22^; also, good compliance was found in India^18^ and Nigeria^5^. These findings were expected to be higher due to the high education level of respondents and proper antenatal teaching on the use of prenatal vitamins, as was observed onsite.

Despite the fact that compliance was observed to be higher with higher education level, the results were not statistically significant. Most of the respondents had tertiary education, and it was expected that education would markedly improve a persons’ outlook and understanding of the health benefits of prenatal vitamins. The findings contradict those of some studies where education was identified as a strong determinant of compliance to iron supplementation in pregnancy^5,13,18^.

In the present study, it was established that there is a statistically significant association between the number of children and compliance with routine medication. Compliance was found to decrease as the number of children increased, corroborating the findings of a study where multigravida women were less likely to comply with iron and folic acid supplements^19^. This may be because of complacency that comes with previous positive experiences and familiarity with pregnancy routines. Multiparous women may feel that there is no need to take these medications, since previous pregnancies and deliveries were without complications, not realizing that each pregnancy is unique. Hence, there is a need for intervention, as past uneventful pregnancies and deliveries do not ensure future healthy ones.

Similarly, a statistically significant association was found between the level of income and compliance with routine medication. Respondents in the lowest income group were the most compliant. This finding is unexpected as one would have thought that a higher income translates to higher purchasing power for the medication and good compliance. However, it could be that those in the lowest income group comply with routine medication in order to avoid adverse events in pregnancy and delivery or congenital deformities in the children, which will be more expensive to manage. This negates the findings in Enugu, as well as in India and Malawi^5,18,20^.

The major deterrents to compliance with routine antenatal medication, as reported by the respondents, were forgetfulness (27.2%) and vomiting (25.7%). These findings, therefore, support that interventions focused on providing reminder messages for pregnant women should be developed and implemented by hospital administrators and health workers as posited by some authors^23^. This view was supported by a study which revealed that reminders such as a diary, mobile phone and calendar pack to take the treatment could improve compliance^5^. They also recommended taking medications with food and counselling on the side effects, as measures to reduce side effects, thus enhancing compliance^5^.

Other factors that influenced compliance to antenatal medication among the pregnant women were non availability of the medication. There would be an improvement in the level of compliance of pregnant women if health workers ensured that the medication is made available without delay to the women. Financial challenges to purchase the medication and lack of support persons were also factors that influenced compliance in this study. These findings, therefore, call for support for pregnant women in terms of subsidizing the cost of the antenatal medication by policy makers and encouraging husbands and significant others to support and encourage their partners. Furthermore, dislike for the colour, shape, taste or odour of the medication, too many pills to take, side effects, fear of having a big baby, and antenatal complications were also barriers to compliance in this study. On the aspects of side effects and antenatal complications, it is a well-known fact that pregnant women become frustrated with complying with antenatal medications when side effects and complications go unmanaged. However, they should always discuss the side effects with the healthcare workers rather than desisting taking the medications. Most importantly, it was further suggested that all the above factors can be effectively endured and managed when the woman understands that it is only for a while and the gestation will soon be over, considering that it is for the wellbeing of herself and the foetus^24^.

### Strengths and limitations

The heterogeneous sample of pregnant women at different gestational ages and parity paints a picture of what happens throughout the stages of pregnancy irrespective of previous experiences. However, as the data were collected at a point in time (cross-sectional survey), it is possible there may be changes in the experiences of the pregnant women. It was observed during data collection that an addition of a qualitative aspect would have enriched the findings on barriers to compliance with routine medication. Also, self-report may be biased as some respondents may give socially desirable responses regarding their use of medication.

## CONCLUSIONS

It was found that the overall compliance with prenatal medication among the pregnant women was poor. It was also demonstrated that while education level has no significant association with compliance, income and number of children were statistically significant. Insights from these findings support the development of evidenced-based approaches for effective prenatal education that hinges on the benefits of routine nutrient supplementation. The findings provide directives for curbing non-adherence by proactively addressing the pregnant women’s concerns with the medication. Midwives should make it a point of duty to assess the use of routine medication on each visit while constantly emphasising the benefits of compliance and dangers from non-compliance. Grand multiparous women should be taught that the risk for an eventful pregnancy and delivery still exists, so that they do not ignore their routine medication on the basis of previous experiences. Since forgetfulness and vomiting caused by the medication were chief barriers to compliance with routine medications, midwives should support pregnant women to set reminders for taking their medications and alternative dosage forms or products that will not induce vomiting should be prescribed. Targeted counselling on side effects and ways to manage or cope with them should also be reinforced by midwives. Policymakers within the institution and in the maternal and child unit of the health management boards can equally enforce instant procurement of routine medication at the point-of-care and medication reviews on follow-up. This will motivate pregnant women to adhere to vitamin supplementation.
